# Increased fascicle length but not patellar tendon stiffness after accentuated eccentric-load strength training in already-trained men

**DOI:** 10.1007/s00421-020-04462-x

**Published:** 2020-08-09

**Authors:** Simon Walker, Joanne Trezise, Guy Gregory Haff, Robert U. Newton, Keijo Häkkinen, Anthony J. Blazevich

**Affiliations:** 1 NeuroMuscular Research Center, Faculty of Sport and Health Sciences, University of Jyväskylä, Room VIV223, 40014 Jyväskylä, Finland; 2 School of Medical and Health Sciences, Centre for Exercise and Sports Science Research, Edith Cowan University, Joondalup, WA Australia; 3 School of Sport, Exercise and Recreation, Southern Institute of Technology, Invercargill, Southland, New Zealand; 4 Australian Centre for Research into Injury in Sport and its Prevention (ACRISP), Edith Cowan University, Joondalup, WA 6027 Australia; 5 Directorate of Psychology and Sport, University of Salford, Salford, Greater Manchester UK

**Keywords:** Eccentric overload, Resistance training, Muscle architecture, Adaptation, Mechanical properties

## Abstract

**Purpose:**

This study examined whether additional external load during the eccentric phase of lower limb strength training exercises led to greater adaptations in knee extensor strength, muscle architecture, and patellar tendon properties than traditional concentric–eccentric training in already-trained men.

**Methods:**

Twenty-eight men accustomed to strength training were randomized to undertake 10 weeks of supervised traditional (TRAD) or accentuated eccentric loading (AEL) or continue their habitual unsupervised (CON) strength training. TRAD and AEL trained 2∙week^−1^ with a six-repetition maximum (RM) session and a ten-RM session. TRAD used the same external load in both concentric and eccentric phases, while AEL used 40% greater load during the eccentric than concentric phase. Tests were performed at pre- and post-training, including: maximum unilateral isokinetic (30°·s^−1^) concentric, eccentric and isometric torques by isokinetic dynamometry, unilateral isometric ramp contractions with muscle–tendon ultrasound imaging to measure tendon stiffness and hysteresis, and resting vastus lateralis and medialis fascicle angle and length measured by extended-field-of-view ultrasound.

**Results:**

After training, both TRAD and AEL significantly increased maximum concentric and isometric torque (*p* < 0.05), but only AEL increased eccentric torque (AEL: + 10 ± 9%, TRAD: + 4 ± 9%) and vastus lateralis (AEL: + 14 ± 14%, TRAD: + 1 ± 10%) and medialis (AEL: + 19 ± 8%, TRAD: + 5 ± 11%) fascicle length.

**Conclusion:**

Both TRAD and AEL increased maximum knee extensor strength but only AEL increased VL and VM fascicle length. Neither training program promoted changes in fascicle angle or changes in patellar tendon properties in our already-trained men.

## Introduction

Short-term strength training interventions provoke robust increases in muscle mass and size (Häkkinen et al. 1985; Narici et al. 1989) as well as the ability to recruit the available muscle mass (Häkkinen and Komi 1983; Knight and Kamen 2001) in previously untrained individuals. Additionally, changes in both muscle structure, i.e. architecture (e.g. Aagaard et al. 2001) and tendinous tissue mechanical properties (e.g. Kubo et al. 2006) have been consistently observed, which may contribute to improvements in muscle function.

With respect to architectural adaptation, increases in fascicle angle have been reported after short-term (i.e. < 16 weeks) strength training (Aagaard et al. 2001; Seynnes et al. 2009; Trezise and Blazevich 2019). This adaptation would theoretically allow for more contractile tissue to attach to the available aponeurosis area (leading to increased physiological cross-sectional area) and for fiber rotation to contribute more to muscle shortening (Brainerd and Azizi 2005), ultimately enhancing force production (Blazevich et al. 2006). Furthermore, increases in serial sarcomere number within the constituent fibers, thereby increasing fascicle length, might also influence force production. Increased sarcomere numbers would influence both the muscle’s force–length and force–velocity properties to specifically enhance dynamic muscle force production (Herzog et al. 1991; Wickiewicz et al. 1983). Nonetheless, observations of increased vastus lateralis and rectus femoris fascicle lengths following short-term strength training have been less consistent (Franchi et al. 2014; Douglas et al. 2018; Mangine et al. 2018; Trezise and Blazevich 2019). One factor that may influence fascicle length adaptation is the contraction mode used during training. In particular, heavy loading during eccentric contractions might stimulate more robust increases in fascicle length, either because of unique aspects of the contraction mode itself or because greater external loads (increasing the total work performed) can be used during the training (Reeves et al. 2009; Franchi et al. 2014). Some researchers consider the eccentric contraction mode itself to be the predominant stimulus in these cases (Franchi et al. 2017; Timmins et al. 2016), leading to the conclusion that eccentric contractions embedded within strength training programs are an important factor leading to fascicle lengthening.

With respect to tendinous tissue adaptation, it is presently unclear how intramuscular connective tissue structures are impacted by short-term strength training in humans. However, detectable increases in (external) tendon stiffness have been observed in the patellar tendon (Kubo et al. 2006; Massey et al. 2018), which may require a critical loading threshold during training (Arampatzis et al. 2007; shown in Achilles tendon), i.e., the most robust findings have occurred in studies utilizing higher (> 70% of maximum isometric or concentric) loading intensities (Kubo et al. 2001; Kubo et al. 2007; Malliaras et al. 2013; Massey et al. 2018; Seynnes et al. 2009). Some evidence suggests that the increases in the patellar tendon stiffness may be predominantly underpinned by changes in the tendon’s material properties, as indicated by increases in Young’s modulus (Malliaras et al. 2013; Massey et al. 2018; McMahon et al. 2018; Seynnes et al. 2009).

Despite these advances in our understanding of the adaptations of muscle architecture and tendon mechanical properties to strength training in previously untrained individuals, limited data exist detailing the training-induced changes in already strength-trained people. It is, therefore, not yet clear whether a ceiling effect might exist. Some evidence has been presented that limited or no changes in fascicle length or angle occur in previously strength-trained individuals (e.g. Douglas et al. 2018; Mangine et al. 2018), although direct assessment of fascicle length (i.e. without the need to extrapolate the length of a fascicle outside the ultrasound imaging area) has not been done in these studies. By contrast, no studies to our knowledge have examined changes in tendon mechanical properties in response to an increase in external load in healthy (i.e. non-clinical) already strength-trained individuals. Therefore, it is unclear whether the continued application of a strength training stimulus is sufficient to promote changes within the tendon even if some changes in loading are imposed.

Total work (regulated in practice by a combination of volume and intensity) and external load (i.e. intensity) independently during strength training appear to be key factors influencing muscle architectural and tendon mechanical adaptations, respectively. Therefore, ongoing adaptation may depend on the provision of greater loads, or volumes, during training. It is not usually feasible to increase loads imposed during the concentric phase of movements in individuals who already attain concentric failure in fewer than ten repetitions, as additional loading would exacerbate fatigue and severely reduce training volume. Nonetheless, significant scope exists to increase loading during the eccentric phase of lifts, given that human muscles can produce greater forces during eccentric actions (Katz 1939) whilst also exhibiting less fatigue (Nishikawa 2016). Accentuated eccentric-load strength training is one method used by weight trainers and athletes to achieve this aim. In this form of training, loads above the concentric maximum are utilized during the eccentric phase of the exercise, whilst the load is immediately reduced to provide a lower (i.e. submaximal) load during the concentric phase. Such training has been shown to be effective in stimulating greater increases in maximum elbow extensor and squat one-repetition strength as well as eccentric knee extension strength in already strength-trained individuals (Brandenburg and Docherty 2002; Douglas et al. 2018; Walker et al. 2016). These strength increases might be expected to be (at least partly) a result of adaptations in both muscle architecture and tendon mechanical properties in this population, if scope exists for such change.

Hence, the purpose of the present study was to compare the effects of 10 weeks of supervised traditional strength training and accentuated eccentric-load strength training on fascicle angle, fascicle length, and tendon mechanical adaptations. We hypothesized that accentuated eccentric-load training would promote increases in fascicle length and tendon stiffness, while no changes would be observed after continuation of traditional strength training. Additionally, we expected negligible change in fascicle angle to occur in either group, given previous findings of a lack of change in already-trained individuals (Douglas et al. 2018).

## Methods

### Study design

The present study used a twin-control design over a 10-week intervention period. A classic control group continued with their normal strength training program without supervision (split-routine). Two groups underwent supervised strength training. The traditional strength training group (TRAD) used the same external load for both concentric and eccentric phases. This group can be considered as the second control group, since they are exposed to the same conditions as the experimental group except for the accentuated eccentric load intervention (Newton et al. 1999). The accentuated eccentric load experimental group (AEL) performed lower body strength training with an additional load during the eccentric phase of each repetition (eccentric load = concentric load + 40%). Subjects in TRAD and AEL continued with their habitual upper body training program unsupervised. Each subject had the time of training standardized throughout the study (± 1 h). Some data sets (maximum strength, muscle cross-sectional area, and volume load during training) from this study have been published previously (Walker et al. 2016, 2017, 2020), and the results for the main strength outcome measures will only be provided in the present paper to provide context to the new muscle–tendon outcome data.

Subjects attended laboratory strength test sessions before and after the 10-week study period, which were 6‒7 days before and after the first and last training session, respectively. These tests consisted of maximum unilateral isokinetic followed by maximum unilateral isometric knee extensions. Additionally, unilateral isometric ramp contractions were performed with simultaneous longitudinal ultrasound scanning of the patellar tendon. During all tests, torque and electromyogram amplitudes (EMG) of the superficial quadriceps and hamstring muscles were recorded. In a separate test session (1‒3 days before the strength tests), ultrasound images of vastus lateralis (VL) and vastus medialis (VM) fascicles were captured with the muscles relaxed (lying supine).

### Subjects

As reported in a previous publication (Walker et al. 2016), 28 healthy men completed all study requirements (21 ± 3 years, 177 ± 7 cm, 75 ± 11 kg). Subjects had a strength training background of at least 6 months (2.6 ± 2.2 years, range: 0.5‒6.0 years). The study methods were approved by the Human Research Ethics Committee of Edith Cowan University and adhered to the Declaration of Helsinki.

Subjects were quasi-randomly allocated to one of three groups based on body mass and maximum unilateral isometric knee extension torque strength. Ten subjects completed 10 weeks of accentuated eccentric-load training (AEL group: 21 ± 2 years, 179 ± 8 cm, 76 ± 11 kg), 10 subjects completed 10 weeks of traditional strength training (TRAD group: 21 ± 2 years, 178 ± 7 cm, 78 ± 12 kg) and eight subjects completed their normal strength training in their own facility without supervision for 10 weeks (CON group: 24 ± 4 years, 176 ± 3 cm, 75 ± 7 kg).

### Training intervention

TRAD and AEL trained twice a week (Monday and Thursday or Tuesday and Friday, to allow at least 48 h recovery between training sessions) under the constant supervision of a qualified instructor from the research team. Each week, training session 1 consisted of three sets of 6-RM loads in the bilateral leg press, unilateral knee extension (3 sets for right leg and 3 sets for left leg alternating) and bilateral knee flexion exercises, while training session 2 consisted of three sets of 10-RM loads. TRAD performed the exercises with the same load for both concentric and eccentric phases, while AEL performed the exercises with 40% greater load during the eccentric phase compared to the concentric phase (i.e., eccentric load = concentric load + 40%). In each training session, the loads used elicited concentric failure in at least one out of three sets in both TRAD and AEL, with the investigator assisting the subject to complete the set. Custom weight releasers were used to add the additional eccentric load to the leg press exercise while weight plates were manually added and removed by the training supervisor(s) with the use of a custom-built pin for the knee extension exercise (Walker et al. 2016). Both groups performed the concentric and eccentric phases of the lift with a 2:2 s tempo (i.e., 4 s in total), which was monitored by the investigator. Immediately after each training session TRAD and AEL, the subjects were given a standardized recovery drink containing 23 g of whey protein (8.47 g leucine and 5.08 g isoleucine per 100 g), 3 g of carbohydrate, and 1.6 g of fat (Total + , Vital Strength, PowerFoods International Pty. Ltd., Marrickville, New South Wales, Australia) to maximize the initial protein synthesis response to training and standardize post-exercise nutrition between groups.

### Strength testing

One week after a familiarization session, where the equipment was adjusted to the individual subjects’ anthropometry and they practiced each strength test, maximum unilateral isokinetic and then isometric knee extension tests were performed with the right leg. Testing began with 5 min cycling (70 rpm, 1-kg resistance) on an ergometer (Monark 818E, Monark Ergomedic, Sweden). Subjects were then positioned in an isokinetic dynamometer (Biodex System 3, Biodex Medical Systems, Shirley, USA) and firmly secured with their knee joint aligned with the dynamometer axis of rotation and inelastic straps were placed across the shoulders, hips, thigh, and ankle to minimize extraneous movement. The limits of motion were set so that each subject performed concentric followed by eccentric knee extensions from a 90° knee angle to a 150° knee angle (straight leg and full knee extension = 180°). Three concentric–eccentric warm-up repetitions were performed at an estimated 50% and then 75% of perceived maximum exertion at 30°·s^−1^ before performing two sets of three maximal repetitions separated by 1 min. The velocity of isokinetic actions was chosen to closely resemble the tempo performed during training.

Torque and displacement data were synchronously recorded at a rate of 2000 Hz using LabChart software (version 6.1.3, AD Instruments, Dunedin, New Zealand) for offline analysis. During analysis, torque, and displacement signals were low-pass filtered (20 Hz cut-off frequency; fourth-order Butterworth). The concentric and eccentric phases were identified from the displacement data and the highest instantaneous torque value along with the corresponding knee angle for each action were obtained for further analysis.

Test–retest reliabilities [intra-class correlation coefficient (ICC) and coefficient of variation (CV%)] from this laboratory have already been reported for the variables included in the present study (Trezise and Blazevich 2019). Additionally here, the variability across 10 weeks in the control group, who continued their normal training and thus provide an indication of long-term variability under training conditions, was calculated. The 10-week reliability values for concentric and eccentric peak torques were 0.903 and 4.7% and 0.947 and 5.6%, respectively.

Then, the subjects were positioned into a custom-built isometric dynamometer (Edith Cowan University, Joondalup, Australia). The knee angle was set to 110° with a hip angle of 100° and subjects were secured firmly by inelastic straps across the shoulders, hips, and right ankle. Each subject was allowed 3–5 practice trials following the commands “push as fast and then as hard as you can”. Real-time visual feedback was provided to instruct the subject to rapidly achieve maximum torque and maintain the contraction for 3–4 s. Three trials were performed, with a fourth trial being required if the third trial yielded more than 5% greater torque compared to the previous trials. Loud, verbal encouragement was given throughout each trial and real-time feedback was provided to the subjects. Torque data were sampled and filtered as described for the isokinetic trials. Analysis was performed offline and assessed for maximum torque and the best trial was taken for further analyses. Rapid torque production was assessed by its change over the initial 50 and 100 ms from torque onset, with torque onset determined by visual inspection (Maffiuletti et al. 2016). Ten-week test–retest reliability for isometric torque was 0.949 (ICC) and 4.1% (CV%).

Thereafter, the subjects performed three ramp contractions using similar methods as Massey and colleagues (2018). Briefly, isometric ramp contractions were performed over a ~ 4 s period for ascending torque and ~ 4 s period for descending torque. Visual feedback was provided throughout the test and the subjects were encouraged to follow a gradually rising and falling torque curve. The trial with the highest torque value was taken forward to further analyses. The preceding maximal isokinetic and isometric tests were considered sufficient for tendon preconditioning. All three tests were repeated after the 10-week study period.

### Muscle architecture assessment

**Fig. 1:**
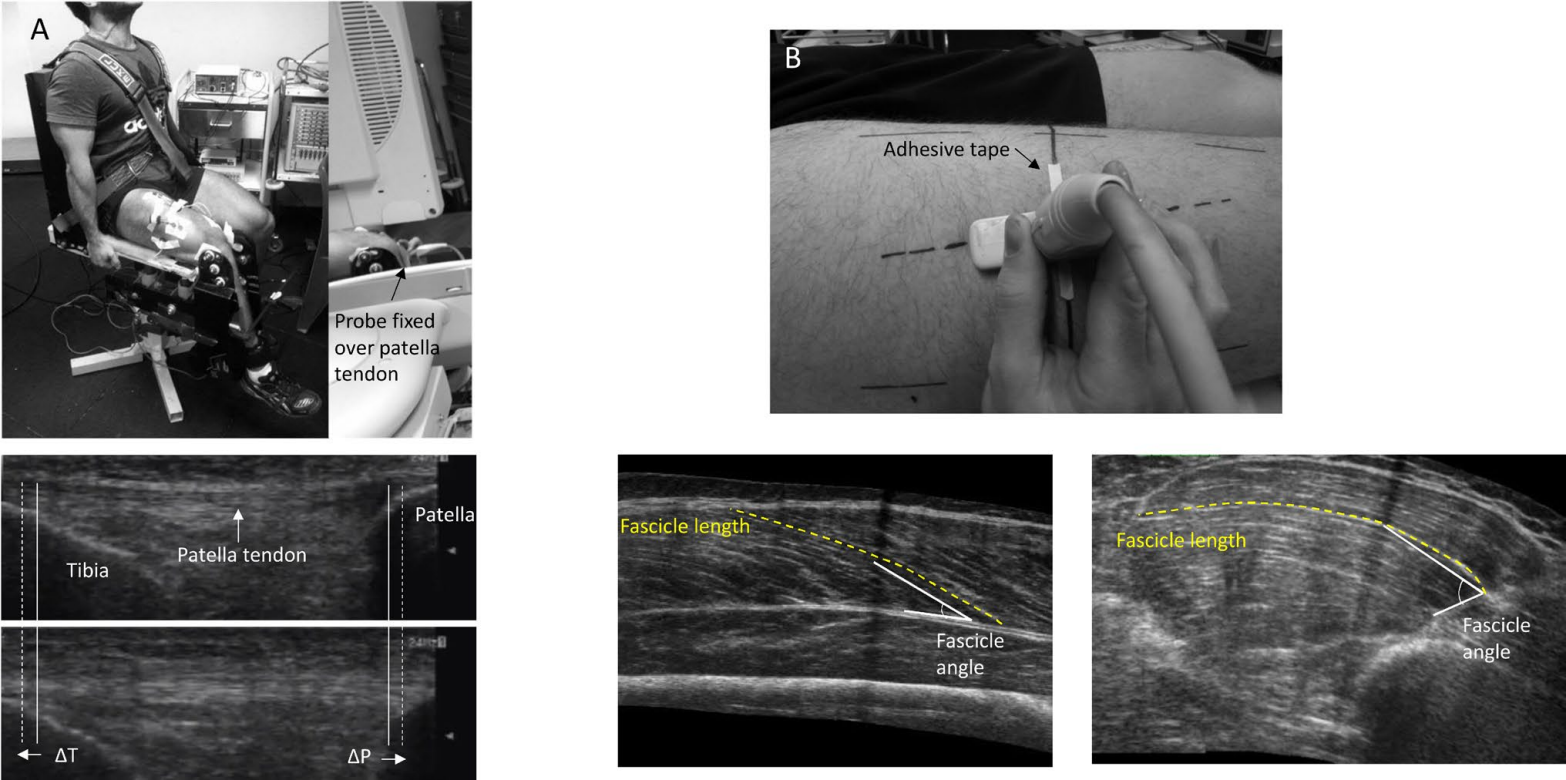
Experimental set up with examples of patellar tendon (**a**) and muscle architecture (**b**) images. Note the curvature of the fascicles for both VL and VM. As fascicle angle was measured to 30% along the length of the fascicle, the values obtained would be smaller than if measured immediately at the deep aponeurosis. However, we observed better reliability when using this method

Muscle architecture assessments followed the procedures of Trezise and Blazevich (2019). In a rested state (1‒3 days before the strength tests and 4‒6 days after the last training session), subjects lay supine on a bench with their knees flexed by approximately 20° supported by a circular wooden rod wrapped in a towel. The subject’s feet were secured together to prevent movement during the measurements. After applying hypoallergenic, water-soluble transmission gel to the skin, VL and VM fascicles and their line of orientation were located at 33% (VM) and 50% (VL) of femur length as measured from the lateral aspect of the distal diaphysis to the greater trochanter (Fig. 1b). Each measurement site was marked by 4-mm wide adhesive tape, which provided a shadow in the ultrasound trace. The fascicle orientations were marked on the skin by a dotted line according to the real-time ultrasound image (Noorkoiv et al. 2010). VL and VM fascicle lengths and angles were assessed using B-mode sagittal-plane ultrasound (model SSD-α10, Aloka Co Ltd, Tokyo, Japan) using a 10-MHz linear-array probe (60-mm width) in extended field-of-view mode (35 Hz sampling frequency). Images were obtained by moving the probe with a slow and continuous movement across the marked line on the skin that followed the line of the fascicles (i.e. approximately towards the patella). Care was taken to minimize compression of the muscle tissue. The mediolateral angle of the probe was adjusted continuously to ensure that the probe was perpendicular to the skin throughout the measurement.

From the four recorded images, the image in which both the fascicle trajectories and muscle aponeuroses were clearest was selected for further analysis; the best image was identified as having two clearly visible fascicles located so that the mid-fascicle length crossed the marker shadow in the image. Fascicle length was determined by manually tracing along the fascicle from the deep aponeurosis to the superficial aponeurosis using Image-J software (version 1.37, National Institute of Health, USA). Fascicle angle was determined by tracing along the aponeurosis to a point 30% along the length of the fascicle using the same software. Each variable was measured three times with the mean of the two closest values taken as the result for that particular fascicle. Thereafter, the mean of the two measured fascicles was used in further analysis. During analysis, the investigator visually inspected pre-training and post-training images in parallel to identify and select (as closely as possible) the same or adjacent fascicles for analysis at both time points with the aid of visible anatomical structures (e.g. relation to bone, tendon, blood vessels, etc.; Blazevich et al. 2007). Test–retest CV% for fascicle length and angle have been shown to be < 3.8% in this laboratory (Trezise and Blazevich 2019). Here, the 10-week test–retest reliability for fascicle length and fascicle angle were 0.822 (ICC) and 6.6% (CV%) and 0.954 (ICC) and 13.5% (CV%), respectively.

### Patellar tendon stiffness assessment

B-mode ultrasound imaging (model SSD-α10, Aloka Co Ltd, Tokyo, Japan) of the patellar tendon using a 10-MHz linear-array probe (60-mm width), image depth of 4.5 cm, and 24-Hz sampling frequency was performed during isometric ramp contractions. After applying hypoallergenic, water-soluble transmission gel to the skin, the probe was held firmly (longitudinally) over the patellar tendon so that the patella and the tibia apex could be visualized throughout the contractions (Fig. 1a). Video recordings captured patellar tendon elongation and shortening and were synchronized to the torque signal.

As is traditionally performed, the activity of antagonist muscles (biceps femoris and semitendinosus) was recorded synchronously with torque data. Here, the intention was to correct the tendon force according to the level of co-activation (Seynnes et al. 2015). However, a recent study (Avrillon et al. 2018) observed that EMG activity of semitendinosus overestimated its tension when acting as an antagonist, potentially due to cross-talk between the hamstring muscles. Furthermore, in another study, the fascicles were observed to lengthen during isometric contractions despite antagonist muscles showing ~ 7‒23% co-contraction (Raiteri et al. 2015). Based on these findings, the use of EMG-torque methods to correct for antagonist muscle forces may increase the error in estimating tendon force by adding noise. In addition to this, no changes were detected in antagonist muscle EMG activities during the ramp contractions relative to their maximum isometric knee flexion activity. Therefore, there was likely no significant change in antagonist co-activation during the knee extensor contractions within subjects. Hence, the decision was taken to not correct tendon force for co-activation.

Longitudinal tendon deformation was measured by tracking the movement of the clear anatomical landmarks of the tibial plateau and inferior border of the patella (Kinovea software, version 0.8.15). This was close to the 1-cm depth at which the ultrasound’s image focal point was set. Both tendon elongation and then shortening were recorded from frames in which joint torque reached 20%, 40%, 60%, 80% and 100% of that recorded during the ramp contractions. After training, tendon elongation and shortening was recorded both at the same absolute (i.e. 20% of the pre-training torque value) and relative (i.e. 20% of the post-training torque value) torque levels. The 100% torque level during the ramp contraction was always above 90% of the maximum isometric torque recorded during rapid, isometric contractions. Analyses were performed on a large screen (133 × 75 cm), and the ramp trial with the highest peak torque and best (subjective) image quality was taken forward to elongation analyses. Each trial was measured twice, with a third measurement taken if the difference between elongation values at each 20% torque increment was greater than 5%. The average of two measurements was used. Patellar tendon force was calculated by dividing the measured isometric knee extension torque by the estimated moment arm length, which was done using the data presented by Bakenecker et al. (2019).

Patellar tendon force–elongation plots were fitted with a second-order polynomial for both the loading and unloading force–elongation curves separately. These polynomials gave *r*^2^ values above 0.93 for loading and above 0.90 for unloading force curve, with a cut-off of 0.9 suggested for acceptability by Seynnes et al. (2015). Stiffness was calculated as the slope of the force–elongation curves from 50 to 100% of the maximum force and to the same force level as measured during the pre-training test session. Hysteresis was calculated as the difference between the area under the loading and unloading curves expressed as a percentage. Ten-week test–retest reliability for tendon properties was 0.779 (ICC) and 29.0% (CV%).

### Statistical analyses

Data are reported as mean ± standard deviation (SD) unless otherwise reported. Tests of normality (Shapiro–Wilk) were run to ensure that the assumptions for parametric statistics were upheld. Repeated measures analysis of variance (ANOVA) was performed on all the measured variables (3 group × 2 time). When a significant *F* value was observed, post hoc tests were performed with Bonferroni adjustments to locate the source of the difference. All analyses were performed using SPSS version 24 (IBM corp., Armonk, NY, USA) and significance was set at *p* < 0.05.

## Results

### Isometric and isokinetic strength

**Fig. 2:**
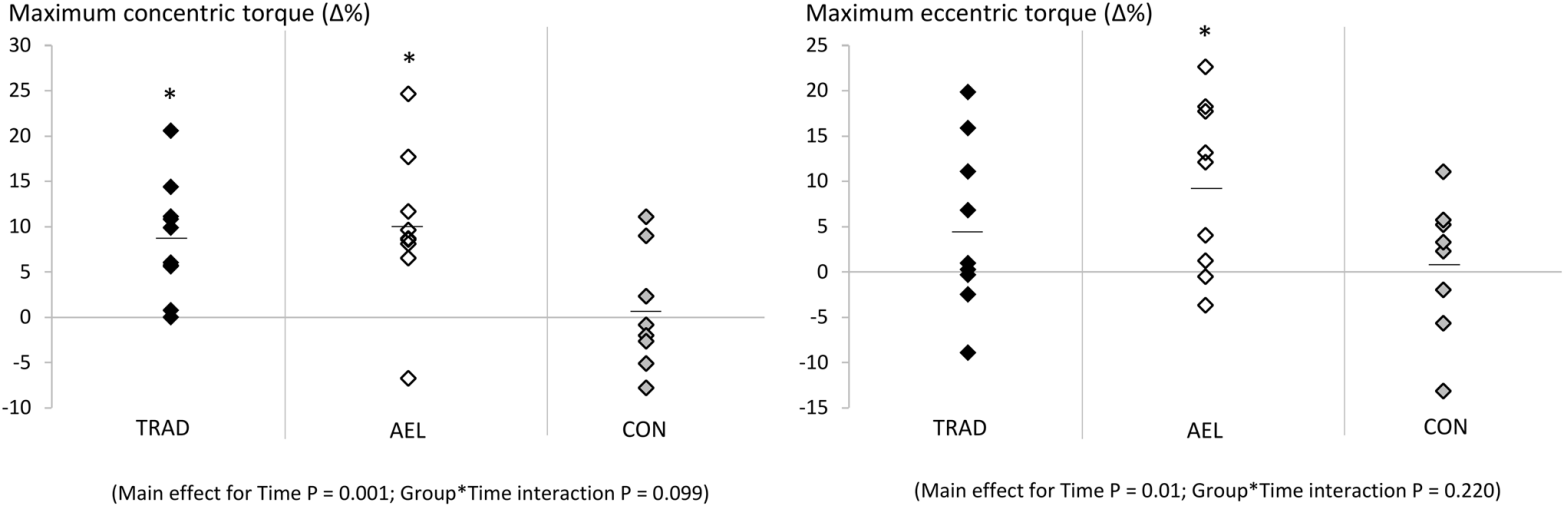
Relative changes (Δ%) in maximum isokinetic concentric and eccentric torque over the 10-week study period for all subjects in TRAD, AEL and CON groups. The horizontal lines represent the group mean. *Significantly different from pre-training values, *p* < 0.05

Significant main effects for time were observed for unilateral isokinetic concentric (*F* = 13.4, *p* = 0.001) and eccentric (*F* = 7.9, *p* = 0.01) torques as well as maximum unilateral isometric (*F* = 25.0, *p* < 0.001) torque. Significant group × time effects were observed for maximum isometric torque (*F* = 7.6, *p* = 0.03) and the change in torque over 0‒50 ms (i.e. RFD) was close to the level of significance (*F* = 3.1, *p* = 0.064). Post hoc analyses revealed that significant training-induced increases occurred in maximum unilateral concentric, eccentric and isometric torques in AEL (Table 1, Fig. 2). TRAD showed significant increases in maximum concentric and isometric torque, as well as a change in torque from 0 to 50 ms (Table 1). No changes were observed in CON. Also, no changes were observed for the angle at which peak torque occurred during maximum isokinetic concentric or eccentric actions.

**Table 1:** Strength, muscle architecture and patellar tendon mechanical properties (mean ± SD) before and after the 10-week study period

	AEL			TRAD			CON		
	Pre-	Post-	Δ%	Pre-	Post-	Δ%	Pre-	Post-	Δ%
Isometric									
MVC (Nm)	277 ± 42	326 ± 56*	17.7 ± 9.8	264 ± 51	291 ± 53*	10.8 ± 11.0	266 ± 48	271 ± 58	1.2 ± 5.2
RFD (Nm)	53 ± 23	47 ± 22	− 7.3 ± 35.5	42 ± 18	56 ± 27*	33.2 ± 30.0	41 ± 23	40 ± 28	13.0 ± 95.3
Concentric									
Peak torque (Nm)	286 ± 41	313 ± 46*	9.9 ± 8.5	274 ± 57	296 ± 58*	8.5 ± 6.2	251 ± 35	252 ± 39	0.5 ± 6.6
Angle at peak torque (°)	103 ± 9	107 ± 5	3.8 ± 8.9	104 ± 9	105 ± 7	− 0.2 ± 12.6	107 ± 5	109 ± 7	3.9 ± 9.6
Eccentric									
Peak torque (Nm)	331 ± 50	361 ± 49*	9.5 ± 9.4	347 ± 54	359 ± 37	4.8 ± 9.3 ±	303 ± 62	307 ± 74	0.9 ± 7.6
Angle at peak torque (°)	107 ± 10	107 ± 7	− 1.3 ± 12.5	107 ± 7	109 ± 7	2.3 ± 8.3	110 ± 5	109 ± 8	− 1.9 ± 10.2
Fascicle angle									
VL (°)	19.1 ± 3.4	19.7 ± 4.0	7.1 ± 16.8	17.9 ± 3.5	18.8 ± 2.7	3.9 ± 13.4	17.3 ± 3.1	17.9 ± 2.4	5.0 ± 17.7
VM (°)	37.7 ±	38.3 ± 10.8	4.3 ± 16.1	37.0 ± 13.5	36.8 ± 8.4	3.5 ± 14.0	35.0 ± 11.0	35.2 ± 10.9	1.4 ± 11.6
Fascicle length									
VL (mm)	73.0 ± 13.2	81.7 ± 9.6*	13.7 ± 13.9	77.0 ± 8.9	78.6 ± 12.8	0.7 ± 10.4	71.3 ± 5.8	69.7 ± 7.3	− 2.0 ± 9.3
VM (mm)	87.9 ± 10.0	104.1 ± 11.5*	18.7 ± 7.8	93.8 ± 8.5	94.4 ± 7.8	4.7 ± 10.7	91.3 ± 7.5	88.7 ± 7.7	− 2.5 ± 8.5
Tendon stiffness									
Absolute (N·mm^−1^)	1437 ± 304	1385 ± 609	− 6.6 ± 26.9	1981 ± 597	1745 ± 710	− 9.5 ± 33.9	2311 ± 903	2422 ± 616	12.3 ± 46.9
Relative (N·mm^−1^)	1437 ± 304	1582 ± 546	8.4 ± 24.6	1981 ± 597	1893 ± 687	− 1.3 ± 30.9	2311 ± 903	1907 ± 616	− 7.7 ± 44.1
Hysteresis (%)	32 ± 16	27 ± 11	− 4.8 ± 41.6	23 ± 13	24 ± 14	12.5 ± 36.1	17 ± 11	16 ± 8	19.7 ± 63.3

Absolute tendon stiffness refers to stiffness calculated over the pre-training submaximal intensities (50‒100% MVC) and, thus, the same absolute external torque levels. Relative tendon stiffness refers to stiffness calculated over the newly measured submaximal intensities (50‒100% MVC) post-training

^*^P < 0.05 versus pre-training

### Resting muscle architecture

**Fig. 3:**
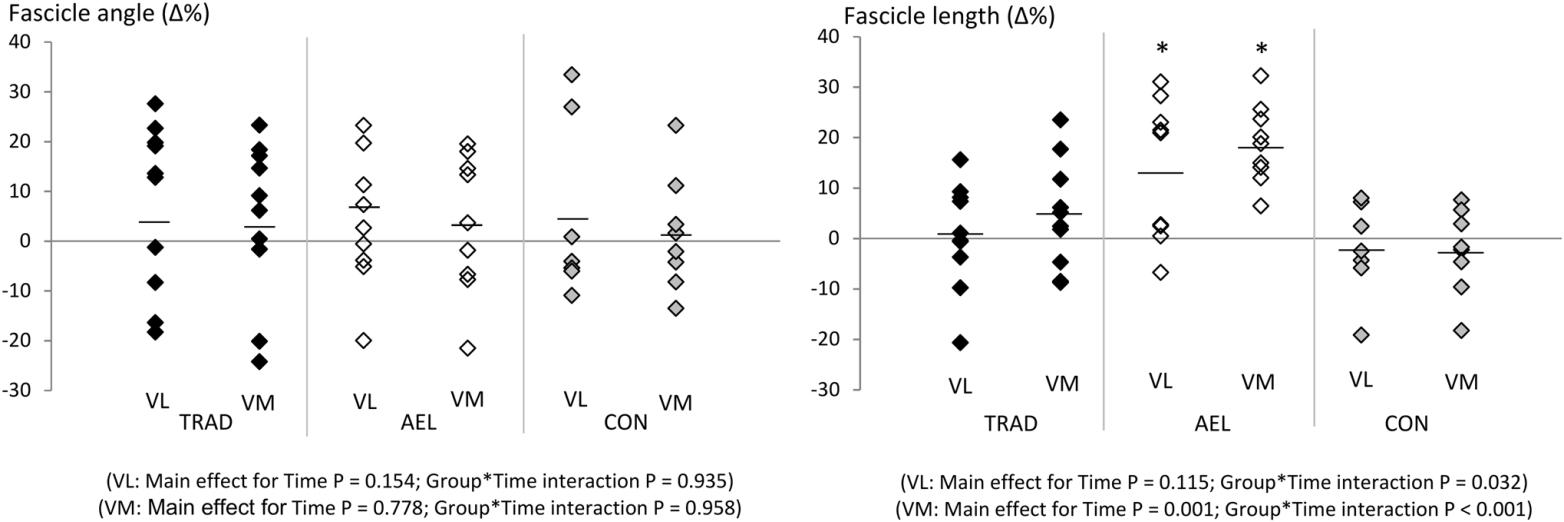
Relative changes (Δ%) in fascicle angle and fascicle length over the 10-week study period for all subjects in TRAD, AEL and CON groups. The horizontal lines represent the group mean. *Significantly different from pre-training values, *p* < 0.05

Significant group × time effects were observed for VL (*F* = 4.0, *p* = 0.032) and VM (*F* = 11.4, *p* < 0.001) fascicle lengths. Post hoc analyses revealed that only AEL showed significant increases in fascicle length after the training period (Table 1, Fig. 3). No group showed a change in fascicle angle.

### Patellar tendon stiffness and hysteresis

**Fig. 4:**
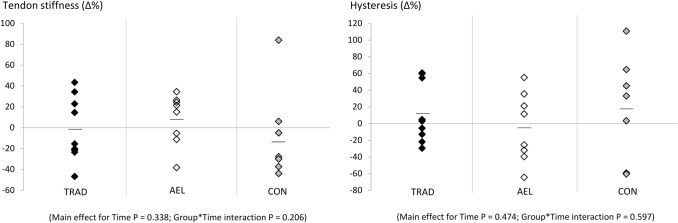
Relative changes (Δ%) in relative tendon stiffness (50‒100% MVC) and hysteresis over the 10-week study period for all subjects in TRAD, AEL and CON groups. The horizontal lines represent the group mean. Note that no changes were observed in absolute tendon stiffness measures either (see Table 1)

No significant main or interaction effects were observed for patellar tendon stiffness from 50 to 100% torque, examined at either the same absolute torque or relative (%MVC) torque as pre-training, or in hysteresis (Table 1, Fig. 4).

## Discussion

The present study compared the effects of 10 weeks of supervised accentuated eccentric-load strength training (AEL) to both supervised (TRAD; traditional training) and unsupervised (CON) traditional strength training on voluntary torque production, vastus lateralis (VL) and medialis (VM) architecture and patellar tendon stiffness and hysteresis in already-trained men. The outcomes included increases in both maximum unilateral eccentric knee extension torque and resting fascicle length in AEL without changes in TRAD or CON (Table 1). However, both AEL and TRAD increased isometric and concentric torque appreciably. For both AEL and TRAD, increases in isokinetic torque occurred in the absence of modifications to the torque–angle relation. Finally, we did not observe statistical changes in patellar tendon stiffness or hysteresis in our already-trained men.

As previously reported (Walker et al. 2016), both AEL and TRAD significantly and similarly increased quadriceps muscle cross-sectional area over the 10-week training period. These muscle size adaptations likely accounted for an influential portion of the observed strength improvements (Balshaw et al. 2018; Maden-Wilkinson et al. 2020; Trezise and Blazevich 2019). Nevertheless, there exists a potential for muscle architectural changes to play a role in increased force production (Trezise and Blazevich 2019). In relation to this, increases in fascicle length were only observed in AEL in the present study (VL: ~ 14% increase, VM: ~ 19% increase). This finding is in line with our hypotheses as well as the results of previous studies (Franchi et al. 2014, 2017) in which higher load eccentric training was shown to evoke greater fascicle length increases than lower load concentric training.

One theory is that serial sarcomere number might preferentially increase with eccentric or eccentric-dominant strength training (Franchi et al. 2017; Timmins et al. 2016). However, little to no empirical evidence of serial sarcomere addition has been shown in humans. Some evidence for serial sarcomere addition after eccentric training has been provided by studies of rat vastus intermedius muscle in response to downhill running (Lynn and Morgan 1994; Lynn et al. 1998). Conversely, preferential effects of eccentric (downhill running) training have not been shown in rat VL under the same conditions (Butterfield et al. 2005) or in response to electrically stimulated eccentric exercise in rabbit tibialis anterior (Butterfield and Herzog 2006) or extensor digitorum longus (Koh and Herzog 1998). Cumulatively, current evidence seems to indicate that other factors, possibly including the muscle length at which force is produced during exercise (Herring et al. 1984; Burkholder and Lieber 1998), may be more important for sarcomere addition; nonetheless, muscle length (and muscle-length-specific force) was not expected to differ between groups in the present study.

If adaptations in serial sarcomere number did occur, then we might expect to observe shifts in the torque–angle relation as well as a change in early rates of force development (which is strongly influenced by the total quantity of series elastic tissue; e.g. Edman and Josephson 2007). However, we found no evidence of shifts in the torque–angle relation. This finding contrasts that of Reeves et al. (2009), who observed a shift in angle of peak isokinetic torque towards longer muscle lengths accompanying a similar (~ 20%) increase in fascicle length, and Blazevich et al. (2007) who observed temporal alignment between changes in fascicle length and shifts in the torque–angle relation after strength training (10 weeks; eccentric and concentric training groups combined) and detraining (14 weeks). Nonetheless, our findings are consistent with other studies showing that changes in muscle activation and, possibly, region-specific hypertrophy largely underpin torque–angle shifts in knee extension (Noorkoiv et al. 2014).

Regardless, increases in fascicle length have been commonly observed in humans after periods of isometric (Noorkoiv et al. 2014), concentric (Blazevich et al. 2007; Franchi et al. 2014) and eccentric strength training (Franchi et al. 2014; Timmins et al. 2016), with some evidence that increases may be greater after heavy-load eccentric exercise (Franchi et al. 2014, 2017). One possibility, therefore, is that increases in fascicle length might be induced by the imposition of fiber/fascicle strains. In contrast to other studies targeting the quadriceps muscles (Franchi et al. 2014, 2017), both total exercise volume and total work performed were similar in AEL and TRAD in the current study (Walker et al. 2017), so training volume/work could not have impacted the results. Therefore, the greater training load may have been a stimulus. Speculatively, the increases in resting fascicle length after chronic high-intensity loading may result from changes in resting sarcomere lengths, reductions in resting muscle tension or increases in end-point tension (e.g. tendinous tissue stiffness), or the longitudinal translation of myofibrils or whole fibers within the muscles (Fridén et al. 1983). While further research is required to understand the mechanism/s leading to resting fascicle length change, it is of practical note that no clear functional enhancements were observed in the present study that might be directly explained by changes in fascicle length (e.g. changes in RFD, shifts in torque–angle relation), as also noted previously (Maden-Wilkinson et al. 2020; Noorkoiv et al., 2014).

It is of interest that the rate of isometric force production increased in TRAD (measured to 50 ms after contraction onset) but not AEL (either to 50 or 100 ms from contraction onset), and that this resulted from a block of training in which rapid force production was not a goal (eccentric and concentric phases were constrained to 2 s each). Despite both TRAD and AEL training under supervision with verbal encouragement, TRAD did not need to pay attention to the additional eccentric load and it being released prior to the concentric phase. This may have led to a training stimulus whereby TRAD could focus on rapid muscle activation during the initial portion of the concentric phase; however, this possibility is purely speculative and further research, with measurements of potential underlying mechanisms, is required to more completely understand these effects and their reproducibility.

Strength training-induced increases in tendon stiffness occur readily in previously untrained individuals (Kubo et al. 2001, 2006; Malliaras et al. 2013; Massey et al. 2018; Seynnes et al. 2009) and this may directly improve force production performance, possibly including RFD (Maffiuletti et al. 2016). However, no changes in either absolute or relative tendon stiffness were observed in our already strength-trained subjects over the 10-week period. Although relative tendon stiffness in AEL showed an average increase of 8%, this increase is smaller than reported in past studies using previously untrained individuals (range 20–80%: Kubo et al. 2017; Malliaras et al. 2013; Massey et al. 2018; McMahon et al. 2018; Reeves et al. 2003; Seynnes et al. 2009) and did not reach statistical significance (*P* = 0.279). Nonetheless, it should also be considered that large variations in tendon stiffness adaptations (partly, a reflection of the test–retest reliability across 10 weeks of continued training, as calculated herein) were observed in the present study, as highlighted by the variances presented in Table 1, which reduces statistical power to detect small training-induced adaptations. Second, absolute tendon stiffness (i.e. tendon elongation measured at the same absolute force values as at pre-training) did not significantly change in either intervention group or when the two groups were pooled, as may have been expected (Table 1). Therefore, no evidence of changes in tendon properties was found using the current methodology in previously strength-trained subjects. Hence, our hypothesis that accentuated eccentric-load training might provide a stimulus for changes in tendon mechanical properties cannot be accepted.

An important point of consideration is that magnetic resonance imaging (or similar technique) was not used in the present study, and moment arm distances were consequently estimated using the method of Bakenecker et al. (2019). Thus, our values will likely differ to those used in other studies, so estimates of tendon stiffness and hysteresis will also differ. As highlighted by Seynnes et al. (2015), moment arm distance differences could lead to 60‒70% differences in calculated tendon forces. Therefore, we cannot directly compare our measurements to others, and cannot draw conclusions as to the effect of previous training experience of our strength-trained men on tendon stiffness. Nevertheless, the values calculated in the present study (mean range: 1385‒2311 N·mm^−1^, Table 1) are similar to those reported previously (e.g. Kobe et al. 2006; Malliaris et al. 2013; McMahon et al. 2017; Seynnes et al. 2009). Furthermore, our within-subject comparisons would not likely be influenced by potential small errors in moment arm as changes in patellar tendon moment arm distance are thought to be negligible even after long-term training (Massey et al. 2018).

Whilst Achilles tendon hysteresis values have been reported between 7 and 37% (Finni et al. 2013), few studies have reported changes in patellar tendon hysteresis after strength training. However, the values of ~ 20–35% obtained by Kubo et al. (2014) and Reeves et al. (2003) are similar to the 16–32% values obtained in the current study (see Table 1), although we did not observe statistical significance. One factor influencing the potential lack of statistically significant changes was the high variability in this variable, which is a consideration for future studies in this area. Such high variance in hysteresis may have been, for example, accentuated by the variation in percentage of MVC attained during ramp contractions (which was closer to 90% in AEL while ~ 95% in the other groups). Nevertheless, secondary analyses on combined data (of TRAD and AEL) to increase sample size, and thus statistical power, also revealed no change. It would be of interest to determine whether other forms of training (e.g. muscle stretching, plyometrics) might influence tendon hysteresis in already trained individuals.

## Conclusion

Strength-trained men increased maximum knee extensor strength over 10 weeks of supervised training using traditional and accentuated eccentric loading programs. However, only accentuated eccentric load training led to increased resting VL and VM fascicle lengths. Neither training program promoted changes in fascicle angle nor changes in patellar tendon properties, including stiffness and hysteresis, in this cohort. These results suggest that fascicle angle and tendon properties may be more resistant to change than muscle strength and mass, and that even a novel increase in loading intensity through an increase in eccentric load may not be sufficient to trigger further adaptation in already strength-trained individuals.

## Abbreviations

AEL: Accentuated eccentric loading
TRAD: Traditional strength training
VL: Vastus lateralis
VM: Vastus medialis
1-RM: One-repetition maximum
RFD: Rate of force development
EMG: Electromyogram
